# Prevent and control cross-transmission of COVID-19 in hospital settings: Lessons learned from a national hospital in Hanoi, Vietnam

**DOI:** 10.7189/jogh.11.03079

**Published:** 2021-06-05

**Authors:** Quang Tuan Nguyen, Thao Anh Hoang, Hai Yen Thị Nguyen, Xuan Co Dao, Van Thanh Do, Van Thanh Dong, Van Giap Vu, Van Minh Hoang

**Affiliations:** 1 Bach Mai Hospital, Hanoi, Vietnam; 2 Hanoi University of Public Health, Hanoi, Vietnam

The COVID-19 pandemic, with the first cases being reported in Wuhan, China by the end of December 2019, has been declared a Public Health Emergency of International Concern (PHEIC) by the World Health Organization (WHO) by the beginning of 2020. Since then, over 112 million people across the globe have been infected with the disease, and more than 2.5 million related deaths, were reported by February 2021 [1]. Vietnam is a lower-middle-income country with a large population of 97 million people. Since the first case of the novel coronavirus being detected in the country in January 2020, Vietnam has experienced three peak-waves of the COVID-19 outbreak during March-April 2020, then July-August 2020, and February-March 2021. Throughout such periods, the country has enforced both on and off nation-wide and regional physical distancing, as well as detected over 2000 positive cases of COVID-19 (among which more than 1600 recovered), with 35 related deaths [2]. At the time of writing this paper, Vietnam has just come off of the third peak of COVID-19 as of March 2021.

Bach Mai Hospital (BMH) is the largest general hospital in Hanoi and Northern Vietnam, providing tertiary care for nearly 3000 inpatient beds and an average of 5000 outpatients per day from across the country. The hospital has 34 clinical centres, institutes, and departments and six para-clinical departments, with over 6000 health care workers and non-clinical staff. Within the context of the COVID-19 pandemic, BMH can be considered a role model of effective rigorous testing, active case finding, and contact tracing; Especially with their experiences of whole-hospital lockdown, sending health experts to support other regions of Vietnam with rising number of COVID-positive cases, and simultaneously staying resilient to provide safety in health care to people in need. In this paper, we share some good practices of BMH in the prevention and control of COVID-19 during the three waves of the pandemic so far in Vietnam. Lessons learned from the hospital could be useful for similar settings in other countries around the world.

Since the beginning of the COVID-19 outbreak in 2020, BMH established two dedicated COVID-19 screening triage system for suspected cases. These were clinics being located separately from other departments of the hospital, one next to the main gate and one near the Center for Tropical Diseases. Screening included general clinical examination, epidemiological data, and having >1 suspicious symptom (fever, cough, shortness of breath) was carried out in these clinics before providing any other services to patients. In case suspected cases were detected, those would be transferred for isolation immediately via dedicated buses to quarantine areas, regardless of not yet having received test results [3].

**Figure:**
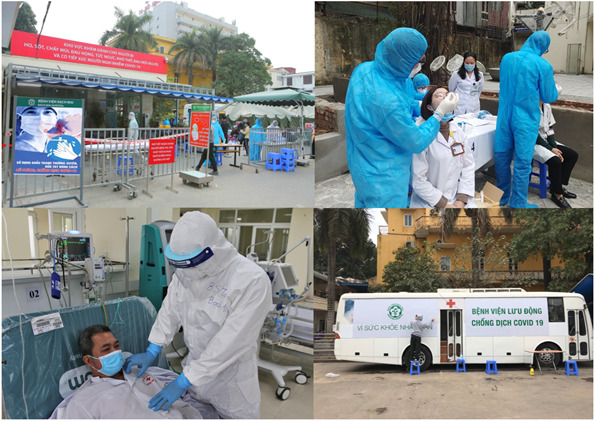
Photo: Bach Mai Hospital in Hanoi, Vietnam battling COVID-19 pandemic (Source: http://bachmai.gov.vn/).

Despite such initial effort during the first wave of COVID-19, cross-transmission at BMH still occurred during March-April 2020. New cases, with unidentifiable sources, were detected in staff of the hospital. BMH took immediate response by isolating affected departments, namely the Center for Tropical Diseases (CTD), National Heart Institute (NHI), BMH Neurology Department, and the hospital catering company. All new admissions, as well as routine visits of patients with chronic diseases were turned down and/or delayed, except for patients with critical conditions. Whole-hospital quarantine of over 7000 people was issued right after just eight laboratory-tests were confirmed positive for COVID-19 on 28 March 2020. Masks for all people being locked down in BMH and protective gear for health providers were required at all times. A high level of infection control was implemented, with spacing patient beds at >2m apart, and allowing only one health provider at a time (unless indicated otherwise) to take care of the patient. Body temperature and medical reporting were also carried out vigorously for everyone going in and out of BMH.

What worth noticing was that, practices of rigorous testing, active case finding, and contact tracing taken by BMH, went beyond the Vietnam Ministry of Health(MOH)’s general guidelines in identifying suspected cases. This meant that, according to MOH’s guidelines, F1 persons were ones having come to close contact with persons with laboratory-confirmed COVID-19 results (also known as F0), and would be under centralized quarantine areas. F2 would be cases of close contact with F1, and would be quarantined and monitored at home; so on with F3 and F4 persons being quarantined at home for 14 days. However, at the time with BMH, definitions of F1,2,3,4 were taken at higher precaution. This meant that since the first case being detected at BMH on 11 March 2020, all people who visited the hospital during 10 March – 20 March 2020 were considered F1 cases, no matter if they have any exposure to COVID-19 confirmed cases. Moreover, PCR nasal and throat testing for COVID-19 was applied to all F1 persons (health staff, patients, caregivers, and visitors of BMH), and both F1 and F2 cases had to undergo quarantine at a centralized area. F3 and F4 persons were self-quarantined and monitored at home or individual private places. By the end of this lockdown, there were a total of 46 confirmed cases of COVID-19 (F0 cases), resulting in over 15 000 people being tested and/or quarantined for having associated with the hospital (F1 and F2 cases), and 40 000 people being traced for coming in contact with the hospital (F3, and F4 cases). This action plan has suggested an effective way of outbreak control, especially in situations involving more vulnerable individuals like hospitals. Owing to quick response and taking precautions, sources of disease and transmission risk were put under control after three weeks, and lockdown at BMH was lifted by 12 April 2020.

During the second wave of COVID-19 in Central Vietnam during July – August 2020, BMH strengthened the continuation of prevention and control measures. These included sterilizing all areas in the hospital, temperature measurement, and medical reporting (if necessary) for staff, patients, and family caregivers. Masks were required at all times to prevent cross-transmission of COVID-19 to other people staying and working at BMH. Simultaneously, since the testing capability in Central Vietnam was still limited at the time, Hanoi’s Centre of Disease Control and Prevention (CDC) appointed the Department of Microbiology of BMH to perform PCR testing 40 000 samples for COVID-19. During such time, the staff at the department had to work intense hours to deliver testing results as soon as possible [4]. BMH also contributed immense support, in terms of providing treatment and testing capability, to Da Nang and Quang Nam provinces – where COVID-19 cases were on the rise. Under the direction of the Health Minister-elect at the time – Nguyen Thanh Long, BMH’s director Nguyen Quang Tuan, along with an expert team of 30 doctors, came to Da Nang to aid the work of outbreak containment [5].

In the risk of the third COVID-19 peak in several cities and provinces across Vietnam, BMH has taken other steps further in their precaution to prevent the pandemic, and also another (possible) lockdown. Currently, all staff, family caregivers, and patients admitted to the hospital were tested PCR for COVID-19; also, the number of caregivers has been restricted to one particular person per inpatient. Two mobile buses for chest x-ray were also set up near the COVID-screening areas [6], and “field hospital” inside BMH’s area was built completely isolated from other departments/ buildings, so confirmed/suspected cases shall be quarantined there immediately, instead of in other temporary isolated inpatient areas as previously. During this wave of January-February 2021, BMH’s experts also organized virtual training COVID-19 prevention and control to health care providers of other hospitals in the Northern regions of Vietnam [7]. The hospital has made vigorous efforts, not only to sustain the provision of care and safety for workers and patients but also to support provincial health facilities where COVID-19 was emerging. BMH has, again, sent health experts to support their collegues at the COVID-19 epidemic in Hai Duong and Quang Ninh provinces [8,9]. Moreover, a “mobile hospital” bus with modern equipment and supplies was also transferred to Dien Bien – one of the poorest-resourced provinces in Vietnam – to tackle a potential epidemic right after just three positive cases being confirmed, as directed by BMH’s director Nguyen Quang Tuan [10].

Collectively, the outlook on BMH playing the key role in this pandemic is positive and hopeful. The highlight of BMH’s practices on outbreak containment of COVID-19 can be boiled down to taking precautions and strict policies to eliminate any possible risk of disease emerging. Also, active contact tracing, vigorous screening, and testing, as well as quarantine adherence, have been the core elements in the preliminary successes and confidence of the hospital in preventing and controlling COVID-19 in Vietnam.
